# Prophylactic Mesh Augmentation of Midline Closure in Patients Undergoing Resection for Upper Gastrointestinal Cancer Reduces the Rate of Incisional Hernia: Results of a Case-Series Study

**DOI:** 10.3389/jaws.2024.13533

**Published:** 2024-11-27

**Authors:** Panagiotis Varsos, Fotios Seretis, Alexis Theodorou, Nikolaos Pachos, Eleni Kitsou, Konstantinos Saliaris, Ioannis Karikis, Dimitrios Theodorou, Tania Triantafyllou

**Affiliations:** First Propaedeutic Department of Surgery, Hippokrateion General Hospital of Athens, National Kapodistrian University of Athens, Athens, Greece

**Keywords:** cancer, prophylactic mesh, esophageal cancer, gastric, onlay mesh

## Abstract

Incisional hernias represent a far more common complication after midline incisions than previously estimated. Patients with upper gastrointestinal tract malignancies represent a group of patients at increased risk for incisional hernia formation after undergoing major surgery. Our prospectively designed study included 50 patients who underwent onlay synthetic mesh augmentation of their midline closure along with closure using the small bites technique. At a 12-month follow-up, no incisional hernias were documented. A significant decrease compared to historical controls was achieved, with few minor complications. Mesh augmentation of midline closure in patients with upper gastrointestinal tract malignancies can significantly reduce subsequent incisional hernia formation.

## Introduction

Incisional hernias are one of the most common complications after surgery, with an increased rate after midline incisions [1]. Risk factors for the development of this complication have been described in the literature, including smoking, obesity and immunosuppression [2]. In patients undergoing abdominal cancer surgery the incidence of incisional hernia development has been reported to be even higher [3]. Incisional hernias present a challenge to these patients’ quality of life, pose a significant risk of intestinal obstruction and strangulation, and are a significant economic burden for health systems [4]. Complex pathophysiological mechanisms including alterations in wound remodeling and collagen deposition have been implicated in the development of incisional hernias [5]. In addition to patient-related factors, surgical technique and use of the appropriate material in abdominal wall closure play a key role [6]. The European and American Hernia Societies recently published their updated guidelines on the closure of abdominal wall incisions [7]. Additional strategies described to prevent hernia development include prophylactic mesh augmentation in high-risk patients [8]. Placement of the mesh in the onlay position or the retro rectus plane are both considered acceptable options. The guidelines recommend the use of permanent mesh as opposed to absorbable mesh, whether synthetic or biological, but the evidence is still very limited and the recommendation in this matter is weak. Initial evidence from prophylactic augmentation of absorbable meshes for the prevention of parastomal hernia [9] or after liver transplantation [10], both considered high risk situations when implanting permanent material, is promising. Phasix™ (Bard-Davol Inc. Warwick, RI, United States) is a fully absorbable monofilament mesh consisting of Poly-4-Hydroxibutyrate (P4HB) (CE-Certified) and is licensed for the treatment of hernias. It is degraded through hydrolysis to the monomer 4-Hydroxibutyrate, a metabolite naturally present in humans that is replaced by the host tissue after 12–18 months.

Oncologic patients are considered immunocompromised [11] and have a higher risk of infection with the use of permanent material. For similar reasons, oncologic patients undergoing major surgery through a laparotomy are considered at high risk for developing an incisional hernia [12]. Patients with upper gastrointestinal malignancies, namely, gastric and esophageal cancer, represent a group undergoing major surgery [13], which is associated with significant morbidity and mortality. The prophylactic placement of mesh in this specific group has not been studied. The purpose of this study is to investigate whether mesh augmentation for the closure of midline abdominal wall incisions during gastrectomy or esophagectomy with an absorbable mesh reduces the rate of incisional hernia formation.

We have conducted a retrospective review of a cohort of patients which, to our knowledge, is the first published real-world data study for incisional hernia prevention with mesh in patients undergoing gastrectomy or esophagectomy for malignancy.

## Methods

We retrospectively reviewed a cohort of patients diagnosed with upper gastrointestinal cancer who underwent radical resection at a tertiary academic surgical unit. The study group consisted of all the patients who had undergone elective gastrectomy or esophagectomy through a midline incision in a 2-year time interval. All patients underwent thoracoscopic esophagectomy.

All midline closures were completed based on a standardized technique using a slowly absorbable, continuous, aponeurosis-only suture using the small-bites technique and aiming for a Suture Length to Wound Length ratio of at least 4:1. A 150 cm USP 2/0 P4HB (Monomax BBraun Surgical S.A.) with a 26 mm ½ circle Taper needle was used for all closures. After the closure of the fascia, a Phasix™ (Bard-Davol Inc. Warwick, RI, United States) mesh was placed in an onlay fashion and secured with 3-0 prolene interrupted sutures. The data recorded included the type and location of the malignancy, type of operation performed, type of neoadjuvant treatment administered, and patient comorbidities. Routine postoperative care followed standardized enhanced recovery after surgery (ERAS) protocols [14]. Patient follow-up included clinic visits and performance of cross-sectional imaging with computed tomography (CT) at certain time points as part of routine postoperative oncological management. CT scans performed at 6 months postoperatively were reviewed for signs of incisional hernia development, seroma formation or other wound-related complications.

## Results

In our retrospective cohort analysis 44 patients were identified in whom an onlay mesh was used to augment midline incision closure. Patient cohort demographics including gender and age were collected. Patients were identified by review of clinical records and all potential identifiers including name and patient hospital identification number were removed. A review of histology revealed adenocarcinoma in 65% of patients (29/44), while 1 patient had squamous esophageal cancer. In 31% of the patients (14/44 patients) no data on histology could be retrieved. The type of operation performed was also retrieved for all patients. A total of 17/44 patients underwent gastrectomy (total, peripheral or extended) and the remaining 27/44 patients underwent esophagectomy (Ivor Lewis thoracoscopic with or without laparoscopic abdominal part of the procedure, McKewn esophagectomy with or without thoracoscopic technique utilization and transhiatal esophagectomy). The majority of patients received neoadjuvant chemotherapy as part of their treatment, namely, four cycles of the FLOT regimen (fluorouracil, oxaliplatin, docetaxel) 21/44 (47%). One patient received radiotherapy in addition to FLOT, while 3 patients received preoperative chemotherapy and radiotherapy, chemotherapy and immunotherapy and chemotherapy, respectively, all of which were not otherwise specified. With regards to nutritional status, preoperative albumin values expressed in mg/dL were available in 41/44 patients (93%) with a mean value of 4.1 mg/dL (range 2.9–4.8 mg/dL). Table 1 contains all relevant information from our cohort, including gender, age, histologic type, type of operation performed, preoperative albumin serum levels, abdominal wall closure related complications and finally the results of the follow-up CT scan with regard to incisional hernia formation rate.

**TABLE 1:** Includes demographics age, gender (male or female), type of operation performed, mean preoperative value of albumin levels expressed in mg/dl and complications relevant to the mesh placement recorded.

Gender	Age	Histologic type	Neoadjuvant treatment	Type of operation	Preoperative albumin level (mg/dL)	Abdominal wall closure related complications	Follow up CT performed
M	73	No available data	No available data	Total gastrectomy	2.9	Seroma-no drain	No hernia
M	78	No available data	No available data	Transhiatal esophagectomy	4.7	Seroma-drain	No hernia
M	81	Adenocarcinoma	4 cycles FLOT	Thoracoscopic Ivor Lewis	4.1	No available data	
M	38	Adenocarcinoma	4 cycles FLOT	Thoracoscopic Ivor Lewis	3.7	No available data	No hernia
M	79	Adenocarcinoma		Total gastrectomy	4.3	No available data	No hernia
M	57	Adenocarcinoma	4 cycles FLOT	Mckeown esophagectomy	4.5	No available data	
M	71	Adenocarcinoma	4 cycles FLOT/Radiotherapy	Mckeown esophagectomy	4.4	No available data	No hernia
M	56	Adenocarcinoma	No available data	Total gastrectomy	4.7	No available data	No hernia
M	83	Adenocarcinoma	4 cycles FLOT	Total gastrectomy	4.2	Seroma-no drain	No hernia
M	76	Adenocarcinoma	No available data	Total gastrectomy	4.3	No available data	No hernia
F	58	Adenocarcinoma	No available data	Peripheral gastrectomy	4.1	Seroma-spontaneous drain	No hernia
M	74	Adenocarcinoma	No available data	Transhiatal esophagectomy	4.6	No available data	No hernia
F	73	Squamous cell	Chemotherapy not otherwise specified	Mckeown esophagectomy	3.8	Dehiscence not clinically evident	No hernia
M	78	Adenocarcinoma	4 cycles FLOT	Extended gastrectomy	4	No available data	
M	58	Adenocarcinoma	Chemotherapy/Immunotherapy not otherwise specified	Total gastrectomy+Right nephrectomy	3.8	No available data	No hernia
M	78	Adenocarcinoma	No available data	Total gastrectomy	4.5	No available data	No hernia
M	60	Adenocarcinoma	4 cycles FOLFOX	Thoracoscopic Ivor Lewis	3.5	No available data	No hernia
F	71	Adenocarcinoma	No available data	Peripheral gastrectomy	4.2	No available data	No hernia
M	66	Adenocarcinoma	No available data	Total gastrectomy	4.2	No available data	No hernia
M	56	Adenocarcinoma	4 cycles FLOT	Thoracoscopic+laparoscopic Ivor Lewis	3.6	No available data	No hernia
F	62	Adenocarcinoma	No available data	Total gastrectomy	4.5	No available data	No hernia
F	73	Adenocarcinoma	4 cycles FLOT	Thoracoscopic+laparoscopic Ivor Lewis	4.2	No available data	No hernia
M	67	Adenocarcinoma	Chemotherapy/Radiotherapy not otherwise specified	Thoracoscopic Mckeown esophagectomy	4.1	Dehiscence not clinically evident	No hernia
F	78	Adenocarcinoma	No available data	Transhiatal esophagectomy	4.2	No available data	No hernia
M	62	Adenocarcinoma	No available data	Thoracoscopic Ivor Lewis	4.3	No available data	No hernia
M	49	No available data	No available data	Total gastrectomy	4.8	No available data	No hernia
M	76	No available data	No available data	Total gastrectomy	3.8	No available data	No hernia
M	41	No available data	4 cycles FLOT	Thoracoscopic Ivor Lewis	4.5	No available data	No hernia
M	72	No available data	4 cycles FLOT	Thoracoscopic Ivor Lewis	4.1	No available data	No hernia
M	65	No available data	4 cycles FLOT	Mckeown esophagectomy	3.3	No available data	
F	63	Adenocarcinoma		Total gastrectomy	4.4	No available data	No hernia
M	60	No available data	4 cycles FLOT	Thoracoscopic Ivor Lewis	4.2	No available data	
F	59	No available data	4 cycles FLOT	Total gastrectomy	4.2	No available data	
M	78	No available data	4 cycles FLOT	Thoracoscopic Ivor Lewis	3.7	No available data	
M	66	No available data	4 cycles FLOT	Total gastrectomy	3.8	No available data	
M	78	No available data	No available data	Mckeown esophagectomy	No available data	No available data	
M	50	No available data	4 cycles FLOT	Thoracoscopic Ivor Lewis	4.4	No available data No available data	
M	54	No available data	4 cycles FLOT	Thoracoscopic Mckeown esophagectomy	No available data	No available data	
M	61	Adenocarcinoma	4 cycles FLOT	Thoracoscopic Mckeown esophagectomy	No available data	No available data	
M	72	Adenocarcinoma	4 cycles FLOT	Thoracoscopic Ivor Lewis	4.1	No available data	
M	60	Adenocarcinoma		Thoracoscopic Mckeown esophagectomy	4.3	No available data	
M	63	Adenocarcinoma	4 cycles FLOT	Thoracoscopic Ivor Lewis	4.2	No available data	
F	84	Adenocarcinoma	No available data	Total gastrectomy	3.9	No available data	
M	65	Adenocarcinoma	No available data	Thoracoscopic Mckeown esophagectomy	3.9	No available data	

Regarding complications related to abdominal wall closure, no wound dehiscence was clinically evident in the immediate postoperative period. Three patients developed a seroma with two of them requiring ultrasound-guided drainage without seroma recurrence. Two patients underwent reoperation due to postoperative complications in which the mesh had to be explanted and not replaced. In one of these two patients, intraoperative wound dehiscence was detected at the time of mesh removal, which had not been clinically evident before. CT scan results at 6- and 12-month intervals are available for 32 out of 44 patients (72%) as of this publication, with no evidence of incisional hernia in any of the patients.

## Discussion

Incisional hernia represents a common complication after major abdominal surgery. A significant body of literature supports the use of prophylactic mesh reinforcements of midline closure in high-risk patients. Results of prophylactic mesh placement in emergency laparotomies report a significant reduction in the rate of fascial dehiscence, at the expense of increased surgical site infections, seroma, and non-healing wound complications [15]. Resorbable biosynthetic mesh placed in the underlay position in clean and clean-contaminated surgical fields appears to reduce incisional hernia development from 22% to 6% [16]. A systematic review and network meta-analysis of randomized clinical trials highlighted that both the onlay position and the retromuscular plane have the best results in reducing incisional hernia formation rates with low numbers needed to treat for the establishment of therapeutic benefit for patients, without a significant increase in perioperative complications [17]. Regarding the use of biologic mesh for midline closure mesh augmentation; a significant body of literature has reported an increased rate of surgical complications [18]. Taking into consideration the aforementioned data along with the recent joint guidelines from the European Hernia Society (EHS) and the American Hernia Society (AHS) [7], we have elected to use synthetic fully bioabsorbable mesh in an onlay fashion for our mesh augmentations. A review of our preliminary results has confirmed the results of other studies, demonstrating both perioperative safety with minimal complications and clinical efficacy for incisional hernia prevention. Consistent with published results in the literature, mesh-related complications were seroma formation in three patients, two of which required intervention, without any reoperations or readmissions for the management of the complications.

Patient-related risk factors have been well described with an emphasis on comorbidities. Patients with gastric or esophageal cancer comprise a group of patients with unique challenges, as they are often malnourished and increasingly receive chemotherapy in the neoadjuvant setting according to medical oncology standards of care. To our knowledge, this is a group of patients that has not been thoroughly studied so far, in terms of incisional hernia prevention.

Limitations of our study include the limited overall number of patients and the incomplete radiologic follow-up of all patients in the cohort. Moreover, the nature of our study, a retrospective cohort review, may introduce a potential selection bias, thus limiting the generalizability of our results. However, the purpose of this cohort study was to examine preliminary results from the application of existing guidelines in a specific group of patients. In essence, it is the capture of real-world data, serving the purpose of continuous improvement of surgical technique and audit of data. When considering the strengths of our study, one needs to consider that it represents the first published study of patients with gastric or esophageal malignancies undergoing surgery with the abdominal part of the operation performed through a midline incision. Importantly, the surgical technique is standardized allowing direct comparison of outcomes with historical controls or patient cohorts from other centers. Another limitation of our study is the lack of comparative data regarding incisional hernia development from our historical controls. Our unit represents a tertiary-level hospital for upper gastrointestinal malignancies with referrals from across the country, making follow-up of these patients very difficult as their care transitions to their local health institutions. More importantly, the implementation of the small bites technique for midline closure of abdominal incision is a practice that has been adopted in the last 2.5 years, making comparisons between a group of small bites technique + mesh augmentation versus closure using other suture techniques without mesh not comparable.

We have confirmed in a cohort of patients undergoing surgery for upper gastrointestinal malignancies that midline incision closure augmentation with mesh is safe, consistent with published results from other patient cohorts representing other clinical entities, for example, colorectal surgery [19].

A recently published randomized control trial (“PRIMA” trial) [20]performed in high-risk patients for incisional hernia development (abdominal aortic aneurysm or body mass index >27 kg/m^2^) confirmed that incisional hernia development after suture-only closure is far more common than previously estimated. Both onlay and retro rectus planes for mesh augmentation appear to be safe and reduce incisional hernia formation rates. However, mesh placement also carries the risk of infectious complications, often necessitating explantation. In our cohort, in all two patients requiring re-laparotomy the mesh had to be removed. Our mesh removal rate was 4%, while data from the aforementioned trial reported a higher rate, although results from a cohort study cannot be compared to results from a clinical trial. A recently published meta-analysis of 15 studies including a total of 2,344 patients failed to prove that surgical site occurrences after mesh augmentation were increased, thus mesh placement continues to be advocated as a risk-reduction strategy for incisional hernia development [21]. The length of the surgery, combined with the obvious need to perform abdominal wall closure in a technically proficient way according to international standards may require a different approach, namely, the development of “closure teams” that would take control of the operation at the end of it, on the one hand to relieve an already fatigued group of colleagues and on the other to ensure with a fresh view that the abdominal wall closure is performed properly and to reduce mesh-related complications such as seroma and hematoma formation.

A very low rate of incisional hernia development was achieved in our cohort by the combination of small bites suturing technique with mesh augmentation. However, the magnitude of the effect of each of these two interventions cannot be estimated. This is an important consideration to make, because if a significant reduction in subsequent hernia development can be achieved by using a proper suturing technique, then the benefit gained from mesh augmentation may be only marginal, making its use potentially not beneficial, especially when considering potential complications. This is a limitation also underlined by the authors of the PRIMA trial because the time period in which the study was conducted was before the implementation of the small bites technique that is now included in the guidelines.

## Conclusion

Mesh augmentation of midline closure with a synthetic bioabsorbable mesh in patients undergoing surgery for esophageal or gastric malignancies combined with small bites closure appears to significantly reduce incisional hernia development over a 12-month follow-up period. No significant complications were encountered. Further studies are needed to identify patient groups that may benefit more from this practice. More specifically, trying to elucidate the exact magnitude of the effect of adding mesh to closure with a proper small bites technique may be a field worth exploring. Moreover, the type of mesh along with the proper tissue plane for its placement in patients undergoing cancer surgery in the upper gastrointestinal tract are both future research questions. To our knowledge, this is the first published real-world data study for incisional hernia prevention in patients undergoing gastrectomy or esophagectomy for malignancy.

## Data Availability

The raw data supporting the conclusions of this article will be made available by the authors, without undue reservation.
